# Using Kalman Filters to Reduce Noise from RFID Location System

**DOI:** 10.1155/2014/796279

**Published:** 2014-01-27

**Authors:** Pedro Henriques Abreu, José Xavier, Daniel Castro Silva, Luís Paulo Reis, Marcelo Petry

**Affiliations:** ^1^Department of Informatics Engineering, University of Coimbra/Centre for Informatics and Systems, University of Coimbra, Pólo II, Pinhal de Marrocos, 3030-290 Coimbra, Portugal; ^2^Department of Informatics Engineering, Faculty of Engineering, University of Porto/LIACC-Artificial Intelligence and Computer Science Laboratory, Rua Dr. Roberto Frias, 4200-465 Porto, Portugal; ^3^Department of Information Systems, School of Engineering, University of Minho/LIACC-Artificial Intelligence and Computer, Science Laboratory, Campus de Azurm, 4800-058 Guimares, Portugal

## Abstract

Nowadays, there are many technologies that support location systems involving intrusive and nonintrusive equipment and also varying in terms of precision, range, and cost. However, the developers some time neglect the noise introduced by these systems, which prevents these systems from reaching their full potential. Focused on this problem, in this research work a comparison study between three different filters was performed in order to reduce the noise introduced by a location system based on RFID UWB technology with an associated error of approximately 18 cm. To achieve this goal, a set of experiments was devised and executed using a miniature train moving at constant velocity in a scenario with two distinct shapes—linear and oval. Also, this train was equipped with a varying number of active tags. The obtained results proved that the Kalman Filter achieved better results when compared to the other two filters. Also, this filter increases the performance of the location system by 15% and 12% for the linear and oval paths respectively, when using one tag. For a multiple tags and oval shape similar results were obtained (11–13% of improvement).

## 1. Introduction

Recognizing the motion pattern of a person or an object in a dynamic environment is a challenging task that has gained visibility in recent years. As such, tracking systems became an interesting research challenge in several areas of expertise, such as computer science, sports, medicine, simulation or robotics, and industrial tracks. Usually, these environments contain several objects (possibly with different motion patterns), which are detected by a tracking system (an introduction to tracking systems is presented below). Depending on the tracking system's nature and the environment where the system is being used, several problems may arise. One of the most troublesome and frequent problem is the introduction of noise in the collected data; for instance, if a vision-based tracking system is used, parts of the environment may be occluded at times; if an intrusive tracking system is used, different materials present in the environment may introduce distortions in the signal, causing noise in the collected data.

Over the years, RFID (Radio Frequency Identification) technology has been used in a large set of tracking systems to perform such tasks. Using a group of tags, these systems are capable of locating an object (human or otherwise) in an environment using distinct approaches, such as simple geometrical rules or learning algorithms [1], among others. NonGaussian noise is normally associated with RFID readers [2]. To reduce such issue, Particle Filters have been used in many research works and presented satisfactory results [3, 4]. Unfortunately, these filters still present an import drawback: their computational costs. Normally, Kalman and Extended Kalman Filters are the key to solve those problems concerning linear [2] and nonlinear motion [5], respectively. However, one question still remains unanswered: what is the best approach for an object that executes a hybrid motion (combination of linear and nonlinear motions), which is probably the most similar to the one carried out by a human? In consequence of that, in this research work, a comparison among variations of the Kalman Filter (namely, the Kalman Filter, the Extended Kalman Filter, and the Unscented Kalman Filter, described below), a known method for noise reduction, was used having an RFID UWB (Ultrawide Band) tracking system as the source for the data, with an associated error of less than 20 cm specified by the manufacturer [6] which, at the time of the experiments, was the best system in the market, when comparing costs and associated error for systems costing up to 20.000 Euro. Comparing to other contexts that presented more than 50 cm of error [5], the major challenge of this work is to detect which is the algorithm that is capable of reducing a 20 cm RFID location system error. For that, both linear and oval patterns were used to evaluate the performance of the filters, using an object moving at constant velocity, equipped with a varying number of tags. Results show that the increase in the number of tags used in the experiments does not translate into an inflation of the registered noise. Also, the Kalman Filter proved to be the best solution for the conducted experiments.

The rest of this paper is organized as follows.  Section 2 describes some works related to the tracking systems area and Section 3 details the methodology used when conducting the experiments and the Ubisense location system.  Section 4 describes the project architecture and, in Section 5, the experimental setup is shown.  Section 6 details the obtained results. Finally, Section 7 presents some conclusion regarding the developed work as well as some lines for future developments.

## 2. Literature Review

In literature, there are many generic tracking systems that emerged over the past few years. These solutions can be divided in two distinct groups: nonintrusive (where there is no equipment on the subject) and intrusive (where sensors or tags are placed on the subject being tracked) [7].

Non-intrusive systems are primarily vision-based systems, where a varying number of cameras are used in conjunction with a set of image analysis algorithms to track the subject. This introduces an additional complexity as well as higher computational requirements if the tracking is to be performed in real-time [8, 9]. In some cases, when there are multiple tracking targets, the visual similarity among these targets, as well as possible object overlapping situations, brings forward additional complexities. Some projects in this area use only one camera for their tracking purposes [10, 11], while others use multiple cameras [12, 13].

Other sensors, such as infrared, may also be used in this group, tracking the thermal signature of live subjects [14]. These techniques have shown promising results in cold environments such as oceans, where the contrast between environment and subject is higher [15], but have some problems, namely its use with objects with no recognizable thermal signature, and also the elevated cost associated with the required equipment.

Intrusive systems encompass a myriad of technologies that can be used to locate and track assets, such as GPS (Global Positioning System) [16], RFID [17] and RFID UWB [18], ZigBee [19], IMU (Inertial Measurement Unit) [20], Wi-Fi [21], or Bluetooth [22], each of which with its advantages and disadvantages that can make each specific technology more suitable for specific scenarios.

The RFID UWB technology is based on the earlier RFID technology, but using a higher frequency band, which translates into higher localization precision, as well as lower power consumption on the transmitter, also allowing for better coverage and coexisting devices in small areas due to more efficient multiple channel access and interference mitigation mechanisms [18, 23]. These systems use a number of receivers and a number of tags, which can be either passive or active. Passive tags do not possess any internal power supply and are only detectable up to approximately six meters from the receiver, while active tags have their own internal power source, offering both reliable detection on a larger scale and higher resilience to occlusion problems caused by possible obstacles in the environment, being detectable up to 100 meters from the receiver.

Table 1 presents a comparison between several technologies, using four performance metrics: cost, accuracy, range, and energy consumption [24]. Values presented in the table are in the range of 0 to 4, 4 being the best classification and 0 being the worst classification. It is also important to state that this comparison table was created according to the collection of information in three distinct studies [7, 17, 25].

By looking at this table, one can see that, in general terms, RFID UWB presents better results than the others (even though its cost is significantly higher than other technologies, such as GPS or ZigBee). Based on the valuable combination of accuracy and range, the UWB technology was selected to perform the experiments, through the use of the Ubisense Real-time Location Systems (RTLS) (more information available at http://www.ubisense.net/en/products-and-services/rtls-products.html); see Section 3.4 for a more detailed description of this solution.

## 3. Used Methodologies and Technologies

In this work, the Ubisense RTLS is used as a location system and three filters were also used to reduce the system noise. These filters as well as the Ubisense RTLS are presented below. 

### 3.1. Kalman Filter

The Kalman Filter (KF) is an optimal estimator that consists on a set of mathematical equations that infers the state of a discrete-time linear process from indirect, inaccurate and uncertain observations [26]. It is considered optimal because, when affected only by white Gaussian noise (normally distributed with mean zero and standard deviation *σ*), the KF minimizes the mean square error of the estimated parameters. The state *x* ∈ *R* ^*n*^ of a discrete-time process is modeled by the linear stochastic difference equation with a measure *z* ∈ *R* ^*n*^:

(1) $$\begin{array}{c} x_{k}=Ax_{k-1}+Bu_{k-1}+w_{k-1},\\ z_{k}=Hx_{k}+v_{k}, \end{array}$$

where *A* is the matrix that relates the state at the previous time step to the state at the current step in the absence of noise, *B* is the matrix that relates the optional control input to the state *x*, *H* is the matrix that relates the state to the measurement *z*_*k*_, and *w*_*k*_ and *v*_*k*_ represent the process and measurement noise, respectively.

The filter response is derived by using a feedback control that estimates the process state and obtains feedback in the form of (noisy) measurements. Thus, the filtering process can be characterized by a cycle of two main stages, prediction and correction.

In the prediction stage, the filter projects forward (from the time step *k* − 1 to step *k*) the current state ${\hat{x}}_{k-1}$ and the error covariance *P*_*k*−1_ in order to obtain the *a priori* estimate of the state ${\hat{x}}_{k}^{-}$ (2) and of the covariance ${\hat{P}}_{k}^{-}$ (3) for the next time step (note that the super minus indicates the variables were computed *a priori*):

(2) $$\begin{array}{c} {\hat{x}}_{k}^{-}={\hat{x}}_{k-1}+Bu_{k-1}, \end{array}$$

(3) $$\begin{array}{c} {\hat{P}}_{k}^{-}=AP_{k-1}A_{T}+Q, \end{array}$$

where *Q* is the matrix that represents the process noise covariance. To close the loop of the feedback control, the correction stage first computes the gain matrix *K*_*k*_ (4) that minimizes the *a posteriori* error covariance. Next, it incorporates a new measurement *z*_*k*_ into the *a priori* estimate to obtain an improved *a posteriori* estimate of the state ${\hat{x}}_{k}$ (5) and error covariance *P*_*k*_ (6):

(4) $$\begin{array}{c} K_{k}=P_{k}^{-}H^{T}{\left(HP_{k}^{-}H^{T}+R\right)}^{-1}, \end{array}$$

(5) $$\begin{array}{c} {\hat{x}}_{k}={\hat{x}}_{k}^{-}+K_{k}\left(z_{k}-H{\hat{x}}_{k}^{-}\right), \end{array}$$

(6) $$\begin{array}{c} P_{k}=\left(I-K_{k}H\right)P_{k}^{-}, \end{array}$$

where *R* is the matrix that represents the measurement noise covariance.

One of the most important limitations of the KF is that it assumes that the system is linear. Once many practical systems have nonlinear state update or measurement equations, a different filtering approach may be required.

### 3.2. Extended Kalman Filter

One possible approach to deal with nonlinear systems is the Extended Kalman Filtering (EKF). The EKF linearizes the state around the current mean and covariance using the partial derivatives of the process and measurement functions to compute the estimates for nonlinear relationships [26]. Assuming that the relation between the current state to previous state is a nonlinear transition function, it can be approximated using first order Taylor approximation, the predictor stage for the EKF can be expressed as:

(7) $$\begin{array}{c} {\hat{x}}_{k}^{-}=f\left({\hat{x}}_{k-1},u_{k-1},0\right),\\ P_{k}^{-}=A_{k}P_{k-1}A_{k}^{T}+W_{k}Q_{k-1}W_{k}^{T}, \end{array}$$

while the corrector stage can be expressed as

(8) $$\begin{array}{c} K_{k}=P_{k}^{-}H_{k}^{T}{\left(H_{k}P_{k}^{-}H_{k}^{T}+V_{k}R_{k}V_{k}^{T}\right)}^{-1},\\ {\hat{x}}_{k}={\hat{x}}_{k}^{-}+K_{k}\left(z_{k}-h\left({\hat{x}}_{k}^{-},0\right)\right),\\ P_{k}=\left(I-K_{k}H_{k}\right)P_{k}^{-}, \end{array}$$

where *f* is a nonlinear function that relates the state at the previous time step (*k* − 1) with the current time step (*k*), ${\hat{x}}_{k}$ is an *a posteriori* estimate of the state at step *k*, *A* is the Jacobian matrix of partial derivatives of *f* with respect to *x*, *W* is the Jacobian matrix of partial derivatives of *f* with respect to *w*, *H* is the Jacobian matrix of partial derivatives of *h* with respect to *x*, and *V* is the Jacobian matrix of partial derivatives of *h* with respect to *v*.

EKF, however, presents at least two significant shortcomings. The first-order linearization of the state distribution only approximates the optimality of Bayes' rule, and thus can introduce large errors in the true *a posteriori* mean and covariance and lead to suboptimal performance [26]. Another consideration regards to the computation of the Jacobian matrices, which are nontrivial in most applications and often lead to significant implementation difficulties [27].

### 3.3. Unscented Kalman Filter

Systems that are highly nonlinear usually are not well modeled by a first order linearization. Since such systems cannot be approximated using EKF, the Unscented Kalman Filter (UKF) was introduced. Similar to KF and EKF, the UKF also takes advantage of the predictor-corrector cycle. However, while the EKF approximates the state of nonlinear systems by simply performing a first-order linearization of the nonlinear functions, the UKF addresses this problem by using a deterministic sampling approach [27, 28]. This sampling technique is known as the Unscented Transform (UT) and is used pick the minimal set of sample points (called sigma points) around the mean. Therefore, UKF algorithm has an additional step beside the predictor and corrector, which is the selection of 2*n* + 1 sigma points (where *n* represent the dimensions of the state space). These sigma points are then propagated through the nonlinear system, from which the mean and covariance of the estimate are then recovered. The result is a filter that more accurately captures the true mean and covariance with no extra computational complexity. The usage of UT results in approximations that are accurate to the third order for Gaussian inputs and at least to the second-order for nonGaussian inputs. The prediction stage, *a priori* state ${\hat{x}}_{k}^{-}$ and the error covariance *P*_*k*_^−^ can be expressed as

(9) $$\begin{array}{c} W_{i}=\left\{\begin{array}{cc} \frac{k}{n+k}, & \text{if}\;\;i=0\\ \frac{1}{2\left(n+k\right)}, & \text{otherwise} \end{array}\right\},\\ {\hat{x}}_{k}^{-}=\sum_{i=0}^{2n^{\alpha }}W_{i}\chi_{i,k\;|\;k-1}^{\alpha },\\ \chi_{i,k}^{-}=f\left(\chi_{i,k\;|\;k}^{\alpha },u_{k-1},0\right),\\ P_{k}^{-}=\sum_{i=0}^{2n}W_{i}\left[x_{i,k}^{-}-{\hat{x}}_{k}^{-}\right]{\left[x_{i,k}^{-}-{\hat{x}}_{k}^{-}\right]}^{T}, \end{array}$$

while the corrector stage can be expressed as

(10) $$\begin{array}{c} {\hat{z}}_{k}^{-}=\sum_{i=0}^{2n^{\alpha }}W_{i}Z_{i,k}^{-},\\ P_{zk,zk}=\sum_{i=0}^{2n^{\alpha }}W_{i}\left[zi,k-\hat{z}k\right]{\left[zi,k-\hat{z}k\right]}^{T},\\ P_{xk,zk}=\sum_{i=0}^{2n}W_{i}\left[x_{i,k}^{-}-{\hat{x}}_{k}^{-}\right]{\left[zi,k-\hat{z}k\right]}^{T},\\ K_{k}=P_{xz}P_{z}^{-1},\\ \hat{x}k={\hat{x}}_{k}^{-}+K_{k}\left(zk-\hat{z}k\right),\\ P_{k}=P_{k}^{-}-K_{k}P_{y}K_{k}^{T}. \end{array}$$

### 3.4. Ubisense RTLS

The Ubisense RTLS provides a solution for asset location in a specific environment using RFID UWB technology. This solution is composed by a number of receptors (typically four) and several active tags, which can be used to track objects.

The receivers of this system are connected to a local network, in order to communicate with a server computer, which runs the Ubisense Platform and collects data from all receivers. The receivers also possess an additional connection, which is used for synchronization—one receiver is used as a master and all other receivers synchronize their clocks by it.

The receivers use three methods to determine the location of each tag—Time of Arrival (TOA), Time Difference of Arrival (TDOA), and Angle of Arrival (AOA). In order to assess the TOA, it is required that tag and receiver be synchronized; by determining the time it took for the signal to travel from the tag to the receiver, the distance of the tag to the receiver can be calculated. When using three receivers, trilateration of the signal is possible, in order to determine the location of the tag. TDOA requires at least three receivers and that they be perfectly synchronized. By determining the difference between the times at which the signal arrives to each receiver, and by means of multilateration, it is possible to determine the position of the tag. Determining the AOA on the receiver requires it to contain multiple antennas arranged in a known configuration, and to be able to determine the TOA for each of the antennas; by combining all this information, the angle at which the signal reaches the receiver can be estimated. An advantage of this method is that the synchronization between emitter and receiver or among receivers is not a requirement.

## 4. Project Description 

**Figure 1:**
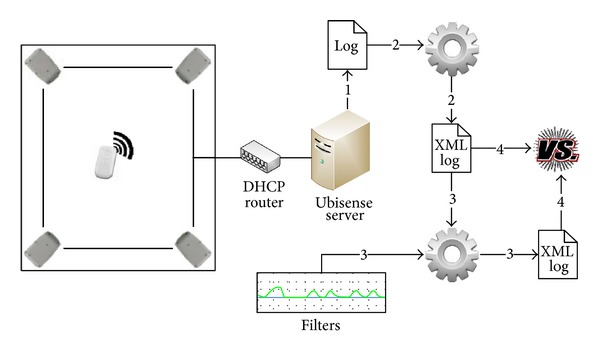
Global project architecture.

The architecture of the project was divided into four steps, as explained below, and following the enumeration in Figure 1.

On step 1, the Ubisense server, which is connected to the four receivers via a network connection, collects the data from the tags and saves it to a log file. This log file contains the location of each tag, which is recorded at a frequency of 40 samples per second (this frequency may be configured for lower values, but in this particular case the maximum possible frequency was chosen to maximize the amount of data collected).

**Figure 2:**
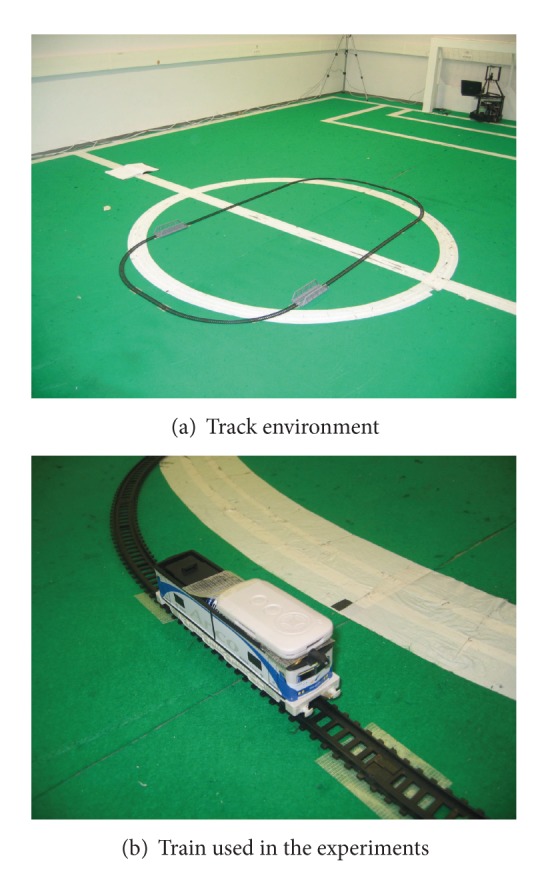
Track and train used in the experiments.

In this step, two different experiments were conducted—one using a linear maneuver and another using an oval one. These maneuvers were attained by using a miniature train moving at a constant speed over a set of tracks disposed in either a linear or oval shape, as can be seen in Figure 2(a). This allows for the position of the train to be determined at each moment with great accuracy. The track was located in an empty room of approximately 12 × 8 m, with a minimum presence of foreign objects (thus attempting to reduce noise in the environment). Also, and in the case of the oval maneuver, the number of tags used varied between 1 and 4.  Figure 2(b) shows the train used in the experiments, with one tag visible on top of it. For each experimental setup, 20 independent tests were performed, as to dissolve the influence of possible outliers. Also, and since the miniature train is powered by batteries, these were replaced every 20 experiments (as to avoid a deterioration of battery performance between experiments).

On step 2, a small application has been developed, as to process the log files generated in step 1, producing files with the data in a standardized format, in this case using a user-defined XML dialect. The format of the produced file follows the Schema specification shown in Listing 1, containing data for a number of tags. Each line of the log contains information regarding one active tag, namely, the identification of the tag and its position (given by three-dimensional Cartesian coordinates) at a given moment.

On step 3, three different filters are applied to each log file, producing new log files. These filters are the Kalman, Extended Kalman, and Unscented Kalman filters, already described in Section 3.1. These filters were developed using MATLAB (more information about MATLAB at http://www.mathworks.com/products/matlab/), and exported to a net component using MATLAB Builder NE (more information available at http://www.mathworks.com/products/netbuilder/).

On step 4, the original log file and the filtered log files are compared using two different metrics—mean squared error (MSE), and maximum squared error (MaxSE). Equation (11) illustrates the MSE metric, where *X* represents a given tag, $\hat{f}$ represents the tag location at a given instant *i* as given by the log file, and *f* represents the actual tag location at a given instant *i*:

(11) $$\begin{array}{c} \frac{1}{n}\sum_{i=1}^{n}{\left(\hat{f}\left(X_{i}\right)-f\left(X_{i}\right)\right)}^{2}. \end{array}$$

**Figure 3:**
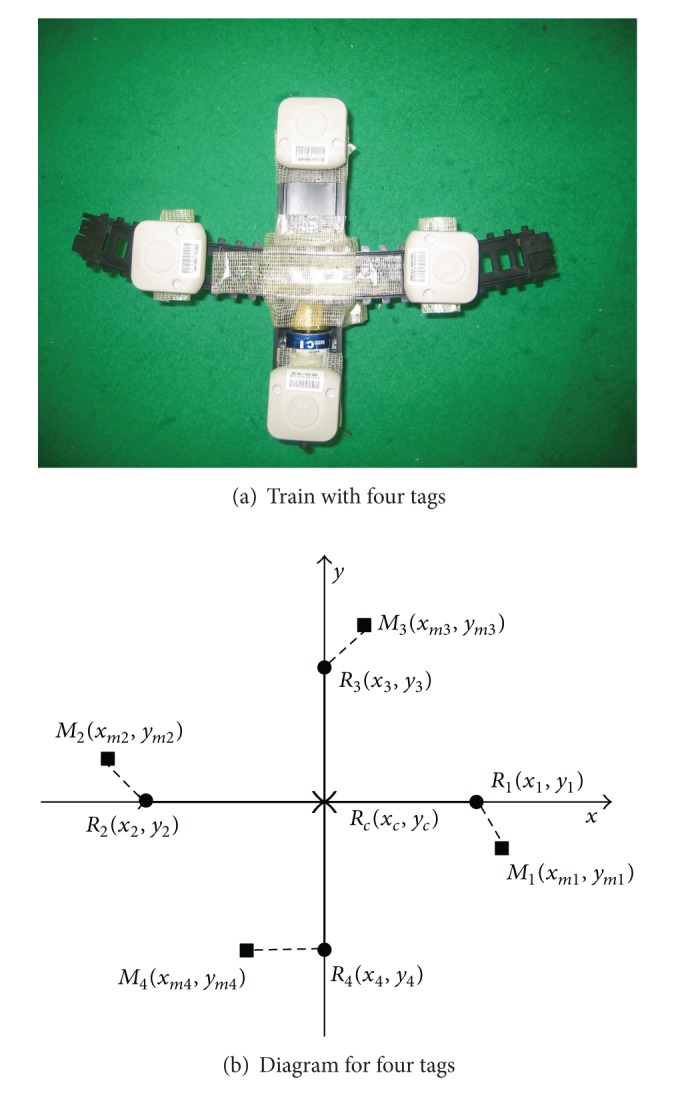
Train and diagram with four tags.

In the scenario of multiple tags being used to track the train (as illustrated in Figure 3(a) for the case of four tags), an additional preprocessing step is performed, determining the centroid of the tags being used. This process is depicted in Figure 3(b), where two distinct types of variables are used, *M*_*i*_ represents the tag position as present in the log files *R*_*i*_ represents the actual tag position; additionally, *R*_*c*_ is the actual centroid of the tags being used. In this approach, the filters were applied to each tag, and only in step 4 is the centroid determined, based on the filtered *M*_*i*_ values.

## 5. Experimental Setup

The Ubisense configuration is a two-step process that can be divided into hardware installation and calibration.

During the experiments, the simplest system configuration for hardware installation was chosen, which consists of monitoring an open rectangular area (clear of obstructions) of 12 × 8 m. The four sensors were mounted at the corners of the area, at 1.6 meters high, and pointed towards the floor in the middle of the space, in order to maximize the line of sight across the tracked space. Once positioned, it becomes necessary to connect the sensors to the Ubisense Server Platform by connecting the sensor network cable to the Ethernet switch. The Ethernet cable provides the sensors not only the ability to communicate with the server, but also the power source required for their operation. At this point, one sensor is configured as a master (and the other three as slaves), by connecting a temporal synchronization cable from each slave to a synchronization port on the master sensor.

As for the calibration process, two steps are required. The first step consists in assigning an IP address of the sensors using a standard DHCP sever. However, since the switch used in the experiments did not provide with a DHCP server, a free ware application (information regarding the used system can be found at http://www.dhcpserver.de/) was used to implement this feature. Next, the sensors need to be added to the server software platform using their MAC addresses. Sensors are them configured by assigning their (*x*, *y*, *z*) position (according to the Cartesian coordinate system) with at least a 5 cm accuracy and a rough estimate of its roll, pitch and yaw. The second step consists on the calibration of the background noise present in the cell. To perform this operation, tags may be disabled and the noise measured using the Incident Power Plot application. From that, it is possible to determine the most convenient threshold to distinguish between valid UWB readings generated by tags and the background noise generated by external sources. Another important calibration step is the calibration of the sensors' orientation and the cable offsets. This calibration is performed by positioning one tag at a known location in the center of the cell, and informing the system of its measured (*x*, *y*, *z*) position. Next, the system acquires a number of samples and computes the optimal values based on the information previously provided, suggesting the required corrections in the angles and cable offsets. Once all the sensors are calibrated, the system setup is completed.

## 6. Results

The results are shown first for the linear path and then for the oval path. As to supply the Kalman filters with the co-variance matrix necessary for its operation, the covariance of the error of the system was determined, using 100 samples for a tag at a known position, and a value of 0.000607 m was obtained.

### 6.1. Linear Path

For the linear path, and as mentioned before, a straight track (with a length of 4 m) was laid out on the room used for the tests and one tag was secured to the top of the miniature train. The train was positioned on an initial short acceleration track that connects to the 4 m track, as to allow the train to accelerate to standard cruise speed when it reaches the track. The experiment was repeated 20 times, and the results are condensed in Table 2, which shows the asverage values for the mean squared error (MSE) and the maximum squared error (MaxSE) for the experiments (measured in meters).

**Figure 4:**
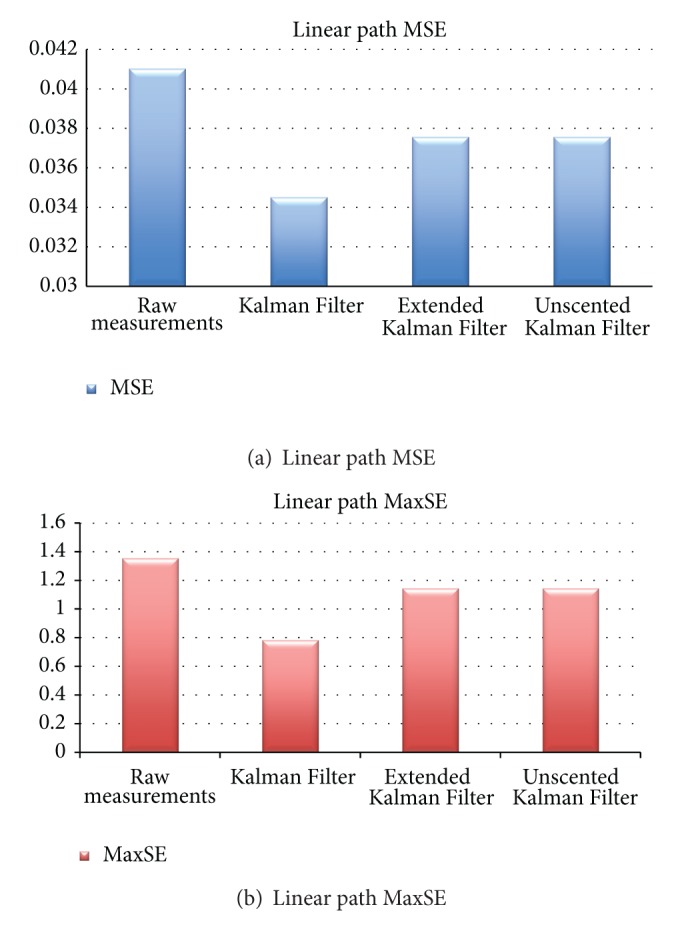
MSE and MaxSE for linear path.

As can be seen from Table 2 and its graphical representation, shown in Figures 4(a) and 4(b), the Kalman Filter introduces an improvement of more than 15% on the MSE, while both the Extended and Unscented Kalman Filters improve results by little less than 8.5%. Regarding the MaxSE, the Kalman Filter enhances the results by 42%, while both the Extended and Unscented Kalman Filters enhance results by only 15.4%.

### 6.2. Oval Path

For the oval path, and as mentioned above, experiments were conducted both with one tag and multiple tags (the results for multiple tags are shown below). In both cases, an oval track (with a perimeter of approximately 10 m) was laid out on the room used for the tests. As to allow the train to accelerate to standard cruise speed, it was placed behind the initial measurement point. The experiment was repeated 20 times, and the results are condensed in Table 3, which shows the Average values for the MSE and MaxSE.

**Figure 5:**
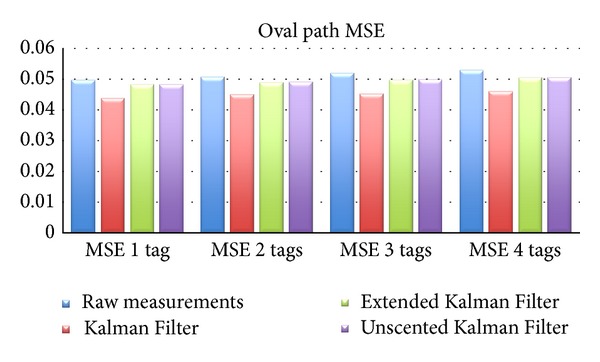
Oval path MSE.

**Figure 6:**
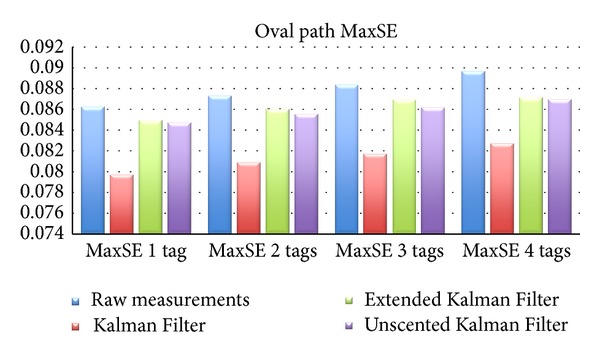
Oval path MaxSE.

As can be seen from Table 3 and its graphical representation, shown in Figures 5 and 6 (results shown alongside results for multiple tags), the Kalman Filter introduces an improvement of almost 12% on the MSE, and an improvement of 7.6% for the MaxSE. The Extended and Unscented Kalman Filters both obtained aproximately 3% improvment for MSE and 1.5% and 1.7% for MaxSE, resp.).

### 6.3. Multiple Tags

Before conducting the experiments with multiple tags, a simple experiment was performed as to determine whether or not having multiple active tags in close proximity would cause signal interference that would increase the error of the system. As illustrated in Table 4, experiments were performed with one and five tags, with results showing that there is no significant difference in using one of five stationary tags.

Experiments were conducted with two, three, and four tags using the oval path (for 20 times). These results are condensed in Table 5, with the values of MSE and MaxSE. For all tags scenario, Kalman Filter performances better than the others. Concerning to MSE, KF improves results by 11–13% while the other filters improve results by 3–4.5%.

Regarding MaxSE, KF improves results by 7-8% while the other two filters improve results by 1.5–3%.

## 7. Conclusions and Future Work

A set of experiments was devised and executed in order to assess the efficiency of Kalman Filters in reducing noise from a location system based on RFID UWB (in this case, the Ubisense RTLS commercial platform was used). For that, experiences involving a miniature train capable of traveling along a path with constant velocity were performed and a comparison study between three variants of Kalman Filters was conducted. It is important to note that the used location system has a low associated error; approximately 18 cm, which turns this research work into an even more challenging project.

Experiments with a linear path show that all variants of Kalman Filters improve the results by reducing the noise introduced by the location system, with the Kalman Filter performing better than the other filters (15%, when compared to the other two, with improvements of about 8.5%).

In what concerns to the oval experimental scenario and using a varying number of tags, the Kalman filter continues to improve the results and the other two used filters present marginal improvements.

Regarding future research lines, one possible direction consists of extending the comparison study to a larger test area with multiple shapes. This requires the acquisition of extra equipment, namely, additional train tracks. Also, it would be very useful if a miniature train with velocity control capabilities was acquired turning the experimental process more flexible and dynamic.

Another possible direction is to execute the comparison study using objects with nonconstant velocity and traveling along a non-preestablished path. This will allow simulating many different real live situations where such a location system could be of use. One such example is the practice of sports (e.g., soccer) where during the game, a coach is a recipient of a huge amount of complex information and because of that tools that can provide automatic soccer performance indicators occupy a major role. However, in this reality it is common that the ball (the most important object in the game) has many occlusion situations, which makes it difficult for a nonintrusive location system (viz. image based) to keep track of it during the entire length of the game. Experiments in an embryonic stage suggest that the RFID UWB can constitute a solution for this kind of sport situations—during these experiments a tag was placed inside a ball traveling along a predefined trajectory in a noisy environment (with many occluding objects), and the results did not show any loss of signal.

Finally, in a more technical level, the developed filter framework can be included in the Ubisense RTLS platform providing a real time object location tracking with reduced noise.

## Figures and Tables

**Algorithm 1:**
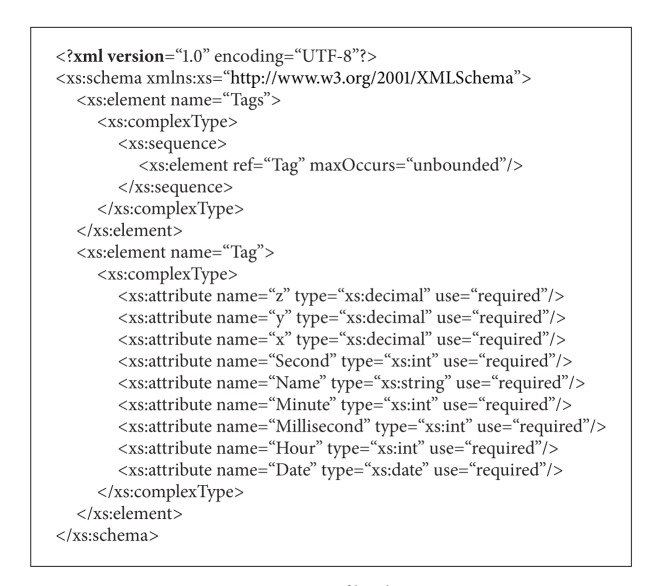
Log file schema.

**Table 1:** Comparison between different tracking technologies.

Technology	Features			
	Cost	Accuracy	Range	Energy consumption
Thermal signature	0	4	1	1
Multicamera	0	4	2	2
GPS	4	3	4	0
IMU	3	2	0	1
Bluetooth	3	1	1	0
Wi-Fi	3	2	2	1
ZigBee	3	3	1	4
RFID	3	2	0	3
RFID UWB	1	4	3	4

**Table 2:** Linear path results.

	MSE	MaxSE
Raw measurements	0.04103	0.07838
Kalman Filter	0.03452	0.04538
Extended Kalman Filter	0.03757	0.06629
Unscented Kalman Filter	0.03758	0.06631

**Table 3:** Oval path results (1 tag).

	MSE	MaxSE
Raw measurements	0.04971	0.08629
Kalman Filter	0.04372	0.07974
Extended Kalman Filter	0.04816	0.08494
Unscented Kalman Filter	0.04824	0.08475

**Table 4:** MSE and MaxSE for one and five tags using Kalman Filter.

	One tag	Five tags
MSE	0.03107	0.03208
MaxSE	0.04173	0.04313

**Table 5:** Oval path results with multiple tags.

	2 tags		3 tags		4 tags	
	MSE	MaxSE	MSE	MaxSE	MSE	MaxSE
Raw	0.05064	0.08734	0.05193	0.08841	0.05284	0.0897
KF	0.04492	0.08091	0.04518	0.08173	0.04592	0.08269
EKF	0.04891	0.08602	0.04973	0.08698	0.05046	0.08713
UKF	0.04907	0.08553	0.04985	0.08619	0.05053	0.08697
